# Cyclosporin A reduces matrix metalloproteinases and collagen expression in dermal fibroblasts from regenerative FOXN1 deficient (nude) mice

**DOI:** 10.1186/1755-1536-6-7

**Published:** 2013-04-02

**Authors:** Barbara Gawronska-Kozak, Heather Kirk-Ballard

**Affiliations:** 1 Regenerative Biology Laboratory, Pennington Biomedical Research Center, Louisiana State University System, 6400 Perkins Rd, Baton Rouge, LA 70808, USA; 2 Institute of Animal Reproduction and Food Research of Polish Academy of Sciences, ul. Tuwima 10, Olsztyn, 10-748, Poland

**Keywords:** Cyclosporin A, Dermal fibroblasts, Matrix metalloproteinases, Nude mice, Scarless healing

## Abstract

**Background:**

Cyclosporin A (CsA), an immunosuppressive agent modifies the wound healing process through an influence on extracellular matrix metabolism. We have compared the effects of CsA on dermal fibroblasts from nude (FOXN1 deficient) mice, a genetic model of skin scarless healing, and from control (C57BL/6 J (B6) mice to evaluate metabolic pathways that appear to have important roles in the process of scarless healing/regeneration.

**Results:**

High levels of matrix metalloproteinases (MMPs) and collagen III expression in dermal fibroblasts from nude (regenerative) mice were down-regulated by CsA treatment to the levels observed in dermal fibroblasts from B6 (non-regenerative) mice. In contrast, dermal fibroblasts from control mice respond to CsA treatment with a minor reduction of *Mmps* mRNA and 2.5-fold increase expression of collagen I mRNA. An *in vitro* migratory assay revealed that CsA treatment profoundly delayed the migratory behavior of dermal fibroblasts from both nude and control mice.

**Conclusion:**

The data suggest that by alternation of the accumulation of extracellular matrix components CsA treatment stimulates the transition from a scarless to a scar healing.

## Background

Cyclosporin A (CsA) is a widely used immunosuppressant for the treatment of autoimmune disorders and to prevent rejection after organ transplantation. However, CsA also causes significant side effects relevant to the healing process. Gingival overgrowth is one of the reported side-effects in 8-70% of CsA- treated patients [1]. This over-growth is characterized by an accumulation of extracellular matrix within the gingival tissue, particularly the collagenous component [1].

Importantly, mammals which are generally considered not to have the capacity for regeneration, display regenerative/scar-free healing in injured gingiva that can be regarded in this respect as a privileged tissue [2,3]. The regenerative capacity of gingival tissue has been attributed to the high levels of collagen and matrix metalloproteinases (MMPs) expression [3,4]. CsA treatment alters collagen turnover in gingival tissue through changes in its capacity to synthesize, degrade and remodel collagens [5,6]. Accordingly, the alterations in metabolism of collagen have been shown to occur in both human gingival overgrowth tissue and in *in vitro* models treated with CsA [1,5-7]. Collagen degradation appears to be modulated through the action of CsA on the MMPs-dependent and MMPs-independent pathways [8]. The gingival fibroblasts from CsA treated patients and animals showed reduced expression of MMP-1 and MMP-3, consistent with conditions that increase collagen accumulation [9,10]. Similarly, MMPs synthesis was decreased in CsA-treated cultured gingival fibroblasts [11].

The anti-regenerative action of CsA treatment was even more evident in the studies by Sicard *et al.*, showing that CsA treatment blocks forelimb regeneration in amphibians, the masters of regenerative potential, in a dose-dependent fashion [12].

Our laboratory has shown that FOXN1 deficient (nude) mice are one of the few examples among mammals that exhibit scarless wound healing. Wounds in both: skin and 2 mm holes in their ears heal without scars by a process that resembles regeneration [13-16]. The post-injured skin of nude mice is characterized by lack of scar, low levels of collagen content, and higher levels of MMP-9 and MMP-13 expression than observed in wild type mice [14,15]. Since nude mice are immunodeficient, in our previous study we tested the hypothesis that the lack of T lymphocytes predisposes them to regeneration/scar-free healing. Accordingly, when we treated control animals with CsA to reduce immune response at post-injured skin area, although T lymphocytes levels were reduced, there was no decrease in scarring of CsA-treated mice. The scar tissues of CsA-treated animals appeared similar or even more prominent (data not shown) than in control untreated mice [14]. Moreover, we subsequently showed that dermal fibroblasts from regenerative (nude) mice expressed higher levels of type I and III collagens, *Mmp-9* and *Mmp-13* mRNA expression and higher MMP enzyme activity than wild type controls, similar to cultured gingival fibroblasts [16]. High levels of MMPs and collagen expression detected in cultured gingival fibroblasts were attributable to scarless healing of gingival tissue, whereas their inhibition by CsA treatment was associated with gingival overgrowth [3,4,9,10].

These observations have led us to the present study in which we test the hypothesis that CsA treatment affects genes of collagens and MMPs more strongly in tissues from mice with regenerative abilities than from non-regenerative wild type mice.

## Results

### Differences in immunophenotype between dermal fibroblasts from nude (regenerative) and B6 (non-regenerative) strains of mice

**Figure 1:**
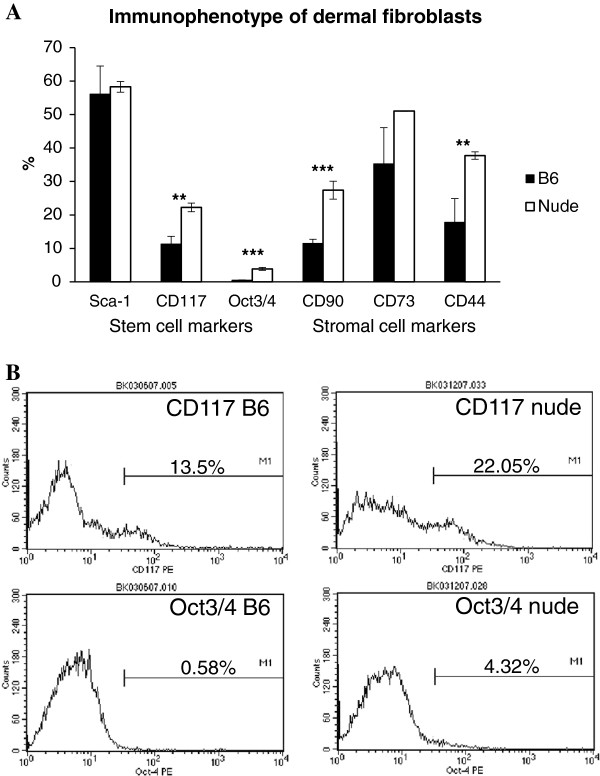
**Immunophenotypic characterization of freshly isolated dermal fibroblasts from nude and B6 mice by flow cytometric analysis. **Cells were labeled with phycoerythrine-conjugated antibodies against Sca-1, CD117, Oct3/4, CD90, CD73 and CD44. (**A**) The data (% of labeled cells) represent the mean of three experiments. Each experiment consisted of a pool of cells collected from three animals. Asterisks indicate significant differences between nude and B6 dermal fibroblasts (** p < 0.01; ***p < 0.001). (**B**) Representative flow cytometric analysis of dermal fibroblasts. The percentage of cells staining positive is indicated on each panel.

Flow cytometric analysis was used to evaluate the phenotypic differences between freshly isolated/non-cultured dermal fibroblasts from the skin of nude (Hsd: Athymic Nude-Foxn1nu) and C57BL/6 J (B6) mice (Figure 1). Cells were examined for the expression of stem cell markers: Sca-1, CD117, Oct3/4 and stromal cell markers: CD90, CD73, CD44. The analysis demonstrated that a high percentage of nude dermal fibroblasts expressed stem cell marker CD117 (22.27% ± 1.27) and Oct3/4 (3.88% ± 0.42), whereas the frequency of B6 dermal fibroblasts were 11.36% (±2.22) of CD117 and did not express Oct3/4 (Figure 1). The frequency of the stromal-associated surface antigens for CD90 was 27.38% (±2.67) and 37.73% (±1.12) for CD44 in nude dermal fibroblasts. The frequency of expression in B6 dermal fibroblasts was much lower for CD90 (11.53% ± 1.18) and CD44 (17.82% ± 7.0). There were no differences in the frequency of Sca-1 and CD73 expression between nude and B6 dermal fibroblasts.

### Effect of CsA on *Mmps* mRNA expression

**Figure 2:**
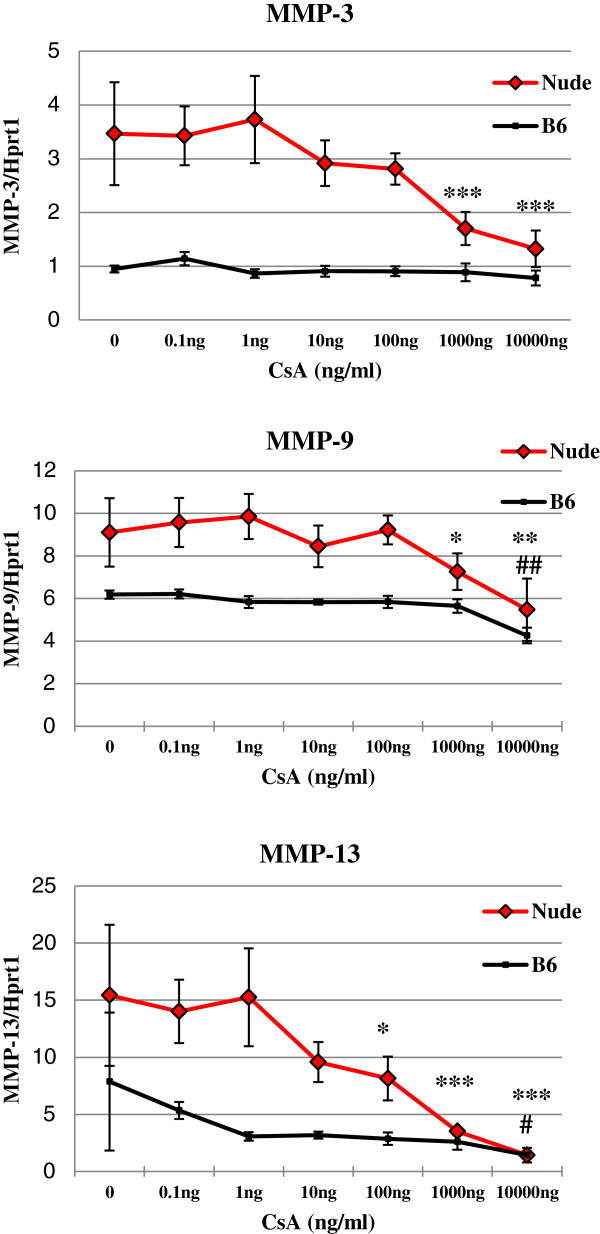
**Cyclosporin A decreases Mmps mRNA expression in dermal fibroblasts.** Quantitative RT-PCR determination of Mmps mRNA levels was analyzed in cultured CsA-treated dermal fibroblasts from nude and wild-type mice. Expression of Mmps mRNA was normalized to levels of Hprt1 mRNA. Triplicate assays were used and experiment was repeated three times each time using dermal fibroblasts isolated from different set of animals (n = 9). Error bars represent SEM; *p < 0.05; **p < 0.01; ***p < 0.001 (nude dermal fibroblasts) and #p < 0.05, ##p < 0.01 (wild type dermal fibroblasts) calculated relative to respective controls, untreated cells (0 ng/ml CsA).

Dermal fibroblasts isolated from regenerative (nude - Hsd: Athymic Nude-Foxn1nu) and from control (C57BL/6 J (B6) strains of mice were cultured under the same conditions (seeding density, collagen coated dishes). Quantitative RT-PCR analysis showed that *Mmp-3*, *Mmp-9* and *Mmp-13* mRNA levels were 3.5; 1.5- and 4-fold higher in nude than B6 dermal fibroblasts (Figure 2). To evaluate the effect of CsA on MMPs expression, cultured dermal fibroblasts from nude and wild type mice were treated for 24 hours with increasing doses of CsA (0-10000 ng/ml). CsA down regulated *Mmp-3, Mmp-9 and Mmp-13* mRNA expression in cultured nude fibroblasts at a concentration of 1000 ng/ml. The largest suppressive effect of CsA was observed for *Mmp-13* mRNA in which levels were suppressed 50% in nude fibroblasts at a low (10 ng/ml) dose of CsA. In contrast, *Mmps* mRNA levels in dermal fibroblasts from wild type mice were not reduced by CsA treatment until the dose of CsA (10000 ng/ml) was 1000-fold higher (MMP-9 (p < 0.01) and MMP-13 (p < 0.05) (Figure 2).

### CsA affects PKC signaling

**Figure 3:**
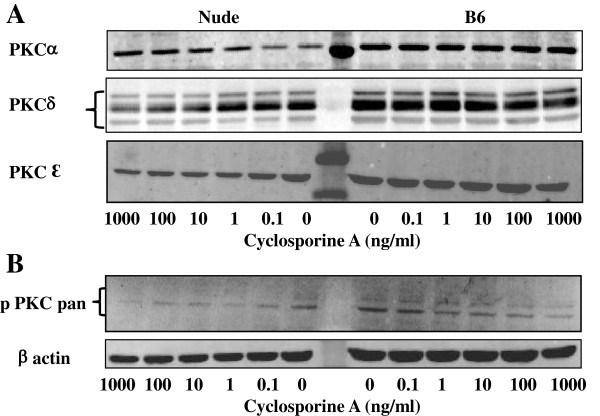
**Cyclosporin A reduces protein kinase C (PKC) signaling in nude dermal fibroblasts.** Primary dermal fibroblast from nude and wild type mice were treated with varying doses of CsA (0.1-1000 ng/ml) for 24 h. Cell lysates was harvested and 40 microgram of total protein per sample was separated on 12% electrophoresis gel. The levels of α, β, ε PKC and phosphorylated-PKC (pan) were determined by immunobloting with specific antibodies. All samples were immunoblotted for ß-actin to assess variation in protein loading. The levels of α, β, ε PKCs (**A**) and phosphorylated-PKC (**B**) are shown.

*In vivo* studies by Watanabe *et al.*, showed that CsA treated FOXN1 deficient (nude) mice overcome some aspects of FOXN1 deficiency, that is, they displayed hair re-growth [17]. In a follow up study they showed that this phenomenon may involve PKC, whose high activity in nude skin is suppressed by CsA treatment [18]. Since CsA reduces levels of *Mmps* in nude dermal fibroblasts to the levels expressed by wild type dermal fibroblasts (see Figure 2) we reasoned that PKC can be a pathway through which CsA regulates MMPs expression. Additionally, our hypothesis was supported by studies demonstrating that the PKC pathway controls MMPs expression in fibroblasts [19]. To examine the effect of CsA on the expression of PKC signaling we used Western blotting techniques (Figure 3). We compared PKC levels in primary cultures of nude and B6 dermal fibroblasts treated with increased doses of CsA. As shown in Figure 3A, levels of PKC α, δ and ε in wild dermal fibroblasts were not affected by CsA treatment. In contrast, in nude dermal fibroblasts a gradual reduction in PKC δ expression occurred with an increasing dose of CsA, whereas PKCα expression increased as CsA concentration raises (Figure 3A). CsA treatment slightly reduced the expression of PKCε in nude dermal fibroblasts (Figure 3A). To determine whether CsA treatment affects PKC phosphorylation, we compared phospho-PKC levels in CsA treated nude and wild type dermal fibroblasts (Figure 3B). Activation of PKC was measured in total cell lysates using anti-phospho-PKC (pan) antibodies. CsA treatment reduced phospho-PKC expression at the higher doses in wild type fibroblasts, whereas in nude dermal fibroblasts the reduction of pPKC pan expression in CsA treated cells was greater and was observed even at the lower doses (Figure 3B). Nude dermal fibroblasts treated with CsA lost higher molecular weight band and showed a gradual reduction in the intensity of the second band. Untreated nude cultures were similar to control and CsA-treated wild type fibroblasts by displaying two bands on the blot, which correspond to the blots provided by antibody supplier (Cell Signaling Technology) (Figure 3B).

### Effect of CsA on type I and III collagens mRNA expression

**Figure 4:**
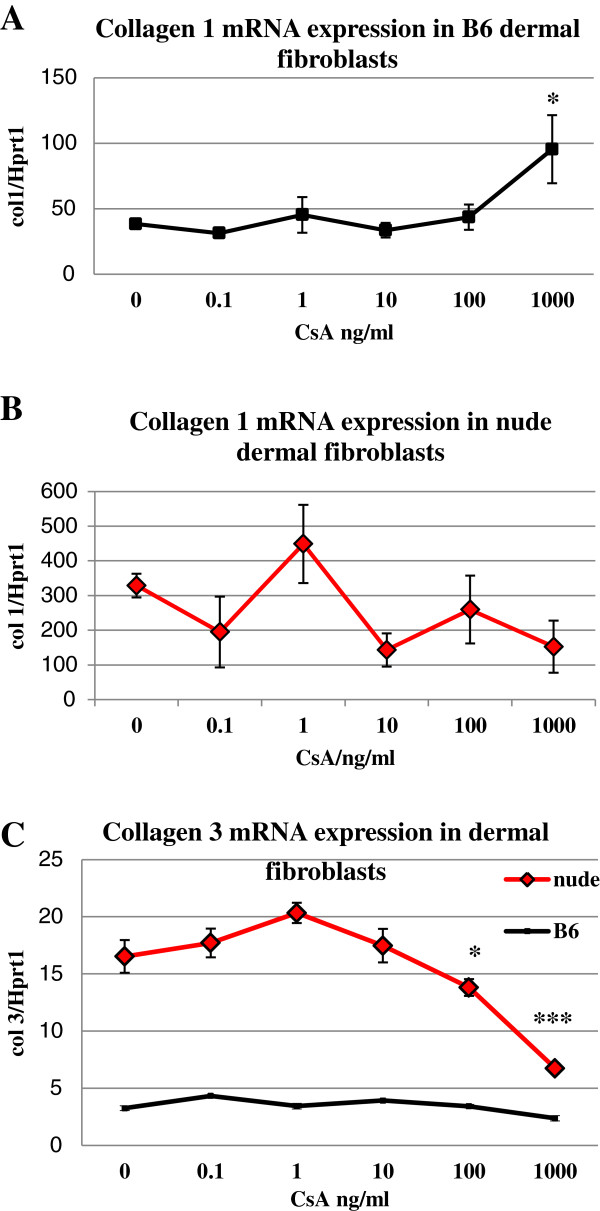
**CsA modulates the expression of collagen I (A and B) and collagen III (C) mRNA in cultured dermal fibroblasts. **Quantitative RT-PCR determination of collagen I and III mRNA levels was assessed in cultured CsA treated dermal fibroblasts from nude and wild-type mice. Expression of collagens mRNA was normalized to levels of Hprt1 mRNA in the reaction. The means and standard errors are shown from three experiments, each performed in triplicate (n = 9); (*p < 0.05; ***p < 0.001), relative to control, untreated cells (0 CsA).

To determine the effects of CsA on collagens we first determined the basal (control) levels of collagen I and collagen III mRNA expression in untreated fibroblasts (Figure 4 A, B and C at 0 CsA). Cultured dermal fibroblasts from nude mice showed 8.5 and 5 fold higher collagen I and collagen III (respectively) mRNA expression than those from wild type mice (Figure 4A, B and C). CsA treatment increased expression of collagen I mRNA 2.5 fold (p < 0.05) in wild type dermal fibroblasts (Figure 4A) but had no significant effect on the levels of collagen expression in nude dermal fibroblasts (Figure 4B). In contrast to collagen I, collagen III mRNA expression in wild type dermal fibroblasts was unaffected by CsA treatment, whereas CsA-treated nude dermal fibroblasts showed a dose dependent reduction in collagen III mRNA expression between 100 ng/ml (p < 0,05) and 1000 ng/ml (p < 0.001) of CsA (Figure 4C).

### CsA delays dermal fibroblasts migration *in vitro*

Since CsA suppresses the elevated expression of MMPs and collagen in nude dermal fibroblasts we asked whether CsA influences the migratory behavior of dermal fibroblasts. We utilized an established *in vitro* wound migration assay [20]. Prior to making the wound, monolayer, confluent cultures of dermal fibroblasts were pre-treated with mitomycin C for 3 hours to suppress further proliferation of the cells. Then, the wounds (scratch) were made and the distance between wounded edges was measured at 0, 6, 12, 24 and 36 post-wounded hours.

**Figure 5:**
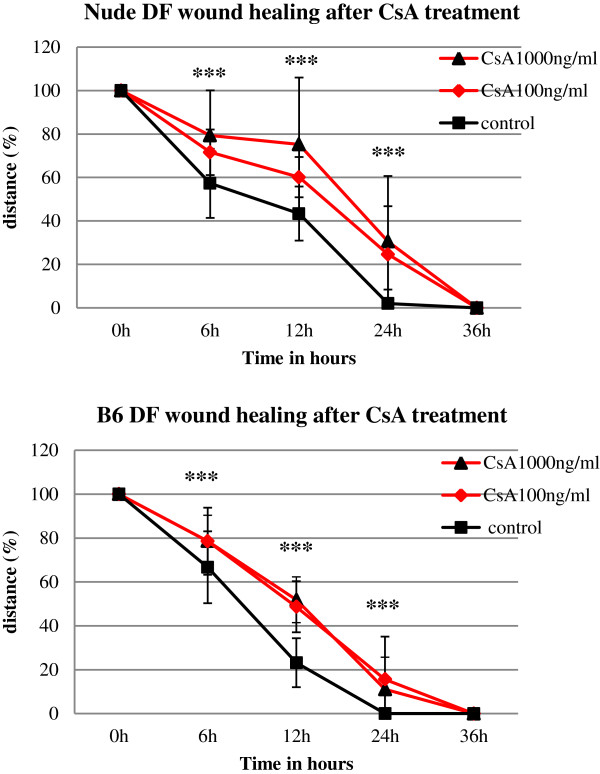
**Cyclosporin A delays dermal fibroblasts wound migration *in vitro*. **Wounded, monolayered dermal fibroblasts from nude **(A) **and wild type **(B) **mice were cultured for 36 hours with CsA at concentrations of 100 ng/ml or 1000 ng/ml. Migration was expressed as a percentage of distance between post-wounded edges of monolayered dermal fibroblasts. Triplicate wells were used and the experiment was repeated three times (n = 9). Error bars represent SEM; ***p value <0.001 calculated from the differences between control and CsA treated cultures at 6, 12 and 24 h.

**Figure 6:**
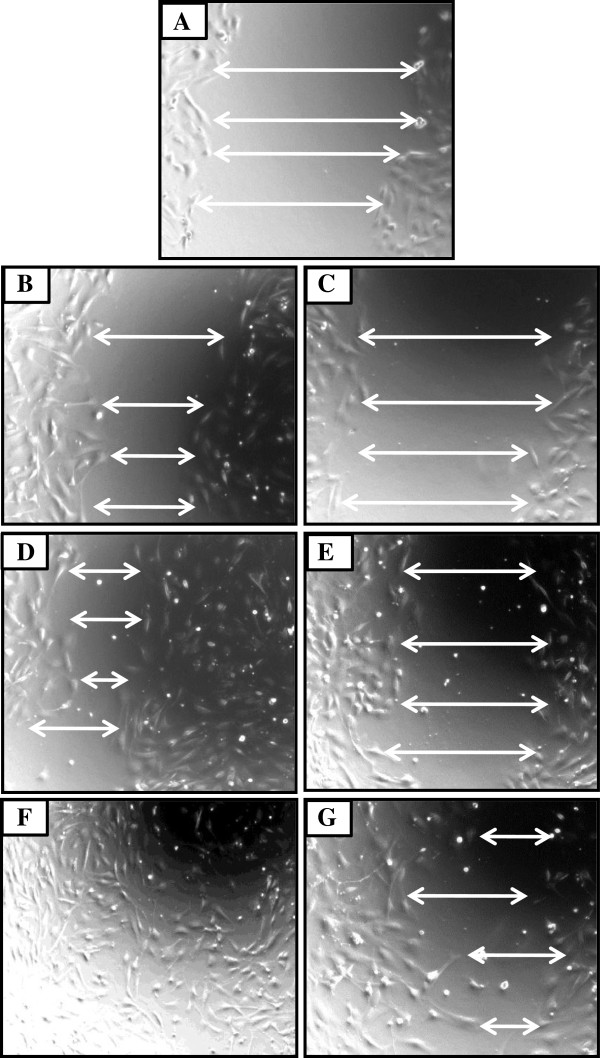
**CsA treatment retards dermal fibroblasts wound healing *in vitro*. **Monolayered dermal fibroblasts were wounded and allowed to heal in the absence **(B, D, F) **or presence **(C, E, G) **of CsA. Representative images from nude dermal fibroblasts cultures were taken at 0 **(A)**, 6 **(B, C)**, 12 **(D, E) **and 24 **(F, G) **hours after scratch. White arrows indicate the distance between post-wounded edges of monolayered fibroblasts.

**Figure 7:**
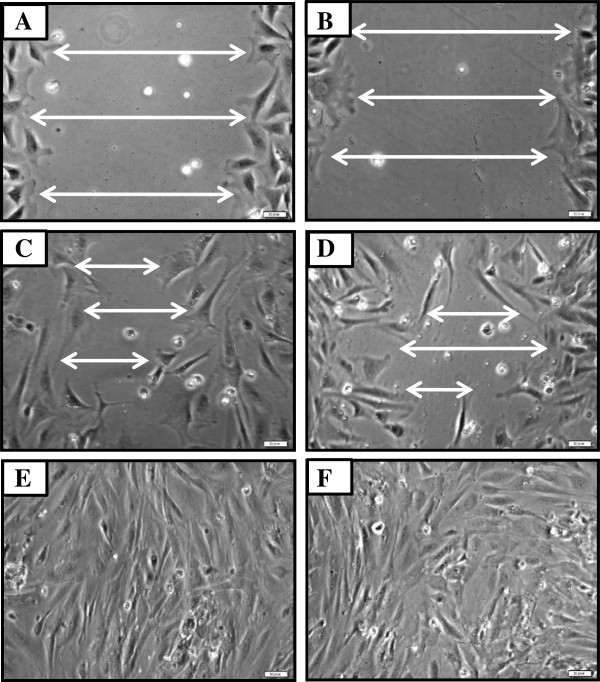
**There are no differences of CsA effect on in vitro wound healing between dermal fibroblasts from regenerative (nude) and non-regenerative (B6) mice. **Wounded dermal fibroblasts from nude **(A, C) **and B6 **(B, D) **were cultured in the presence of CsA. The images were taken at 6 **(A and B) **and at 24 hour **(C, D) **after treatment. Non-treated dermal fibroblasts at 24 hour: **(E)** nude, **(F) **B6.

Overall the data showed that CsA significantly delayed the migration of fibroblasts from both nude and wild type animals (Figures 5, 6, 7). The migratory delay in CsA-treated cells was observed at the 6, 12 and 24 h time-period (Figures 5, 6) with doses of either 100 or 1000 ng/ml CsA (Figure 5).

Wound closure in untreated dermal fibroblasts from both nude and wild type mice occurred in about 24 h [Figure 5; Figure 7E (nude) and F (B6)], whereas open wounds in CsA-treated fibroblasts were still present [Figure 5; Figure 7C (nude) and D (B6)]. CsA retarded wound closure to a similar extent in both nude and B6 dermal fibroblasts (compare Figure 7A and B; Figure 7C and D).

## Discussion

In the present study we have assessed the effects of CsA on dermal fibroblasts from nude (FOXN1 deficient) mice, animals that are capable of healing skin injuries and punched ears in a scar-free/regenerative fashion. Our data show that CsA alters expression of collagens and *Mmps* synthesis and retards the migration of dermal fibroblasts, changes expected to affect the structure of the ECM. Additionally, the data suggest that CsA particularly affects tissues which are privileged for regenerative features, that is, skin of nude mice (present data), forelimb in amphibians [12] and gingival tissue [2].

Immunophenotypic characterization of dermal fibroblasts showed substantial differences between nude (regenerative) and B6 (non-regenerative) mice. Dermal fibroblasts from nude mice are characterized by high levels of stem cells markers expression (CD117 and Oct3/4) that are low (CD117) or absent (Oct3/4) in non-regenerative B6 mice. The expression of stem cell markers in nude dermal fibroblasts may indicate that nude mice possess features of embryonic development in adulthood that are absent in B6 mice and may explain their ability for scarless skin healing. Interestingly, the presence of embryonic and fetal molecular, metabolic and cellular features retained during adulthood was observed in another regenerative strain of mice, MRL [21]. However, from the another point of view, the sole existence of mesenchymal stem cells in adult tissues is not sufficient for regeneration to happen as we showed for mouse ear hole closure [13]. External ears of regenerative and non-regenerative strains of mice contain a population of mesenchymal stem cells (EMSC - ear mesenchymal stem cells) that are capable of differentiating into 4 lineages *in vitro*. Nevertheless, only holes in the ears of nude mice heal with the features of the regenerative process [13]. Regardless whether the stem cell features of nude dermal fibroblasts can account for scarless skin healing, the profiles of these stem cell markers underline the existence of immunophenotype differences between nude and B6 dermal fibroblasts. Moreover, dermal fibroblasts derived from FOXN1 deficient (nude) mice produce higher levels of *Mmp-3, -9* and *13* than B6 wild type. CsA treatment reduced the levels of *Mmps* expression in nude dermal fibroblasts to the levels observed in B6 wild type (control), but had no effect on *Mmps* expression in B6 wild type mice. Our data are consistent with results obtained in other studies on the effect of CsA on gingival fibroblasts. The gingival fibroblasts from CsA treated patients and animals showed reduced expression of MMP-1 and MMP-3 [9,10]. Similarly, CsA treated cultured gingival fibroblasts had a decrease in MMPs synthesis [11,22]. The action of CsA among published studies has varied from suppressive (as described above and in our studies) to stimulative (increase in MMPs and collagen [7]). The observed discrepancies could be due to *in vivo* vs *in vitro* studies. Additionally, the type, origin and state of development of the cells (*in vitro* study) and individual differences among subjects (*in vivo* study) could determine the course of CsA action. The most profound differences in CsA action was observed between gingival and dermal fibroblasts. The synthesis of collagen Iα1 was stimulated in keratinocytes co-cultured with gingival fibroblasts treated with CsA [23] and suppressed in cultured dermal fibroblasts [24]. Similarly, our studies showed that CsA treatment reduced collagen III expression in nude fibroblasts, but expression was unchanged in B6. Simultaneously, CsA increased collagen I in B6 but had no influence on collagen I expression in nude mice (see Figure 4). Higher and sustained levels of collagen III expression characterize scar-free skin healing in mammalian fetuses that is observed during first two trimesters of gestation [25]. Fetuses from last trimester of gestation, as well adult mammals, heal skin injuries with scar formation which is accompanied by higher levels of collagen I expression in postinjured area. Changes from higher to lower ratio of collagen III to collagen I accumulation in postinjured skin tissues is one of the indicators of transition between scar-free to scar-forming healing in mammalian fetuses [25]. Our data indicate that CsA treatment (decrease levels of collagen III in nude/regenerative fibroblasts and increase levels of collagen I in control fibroblasts) contributes to the accumulation of extracellular matrix components that can cause the switch from scar-free to scar forming healing. Accordingly, we propose the concept that CsA may interfere in regenerative (scar-free) healing processes as observed for gingiva and during amphibian limb regeneration [12].

However, an unwanted side effect of CsA treatment, observed in gingival tissue, seems to be beneficiary for unhealing skin wounds [26,27], that is, CsA treatment not only reduced inflammation, but also promoted skin healing in healing-resistant lesions [26,27].

Generally the outcome of the healing process is attributable, among others features, to the MMP/TIMP ratio and the collagen synthesis/degradation rate. Regular healing is characterized by equilibrium between MMP and TIMP which is essential during wound repair [28]. A decrease in MMP-to-TIMP (ie. 1:2) may contribute to increased synthesis and deposition of collagen, leading to pathological (overgrowth) scar formation [29]. On the other hand, an increased MMP/TIMP ratio due to high levels of MMPs and low levels of TIMPs accompany delayed wound healing [28,30]. Similar dynamic changes in MMPs and TIMPs expression was observed during wound healing in diabetic humans and animal models [31,32]. However, increased MMPs expression has also been observed during perfect, scarless healing in mammalian fetuses, gingival tissue and skin wounds in nude mice [3,15,16,33,34]. Thus, changing the balance between MMP/TIMP and collagens synthesis/degradation by CsA treatment can influence the outcome of the healing process.

**Figure 8:**
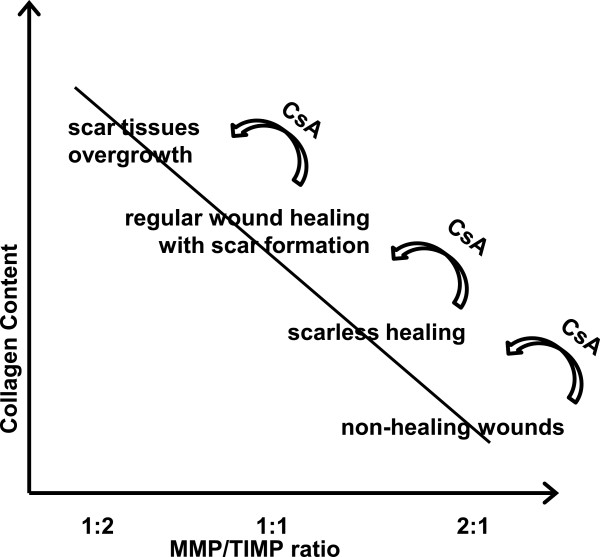
**A model to illustrate how CsA treatment can modulate outcome of the healing process.** CsA contributes to extracellular matrix accumulation through reduction of MMPs expression (MMP/TIMP balance) and modulation of collagen accumulation. In non-healing wounds reducing MMPs expression by CsA treatment promotes a healing process that resembles scarless healing. Scarless healing (e.g. gingival tissue and nude skin) is characterized by a modest elevation of the MMP/TIMP ratio which is shifted by CsA treatment to a balanced MMP/TIMP ratio (1:1) and results in healing with scar formation. Further suppression of the MMP/TIMP and increasing collagen content progresses from regular wound healing with scar formation to the overgrowth of scar tissues.

Accordingly, we propose that CsA treatment may modulate the outcome of the healing process (Figure 8). Application of CsA to non-healing wounds (high levels of MMPs and low levels of collagen) will reduce MMPs expression and stimulate collagen synthesis to promote healing. For scarless healing, requiring higher levels of MMPs and lower levels of collagen, CsA treatment results in the reduction of MMPs and collagen to the levels measured in normal, scar-present healing. For regular wound healing with scar formation, normally observed at a MMP/TIMP ratio 1:1, CsA treatment will shift the ratio towards 1:2 and increasing collagen accumulation characteristic of scar overgrowths. Although the mechanism of CsA action is still unclear, our intention is to turn attention towards the beneficial effects of CsA in treating diabetic skin ulcers and to the undesirable effects of CsA on the hindrance of tissue regeneration.

## Conclusions

We have demonstrated that CsA treatment down-regulates the expression of MMPs and collagen III expression in dermal fibroblasts from regenerative (nude) mice to the levels observed in non-regenerative (B6) mice. Moreover, an *in vitro* migratory assay revealed that CsA treatment profoundly delays the migratory behavior of dermal fibroblasts from both nude and control mice. The data suggest that CsA particularly affects tissues privileged for regeneration and stimulates the transition from scarless to scar healing.

## Methods

### Isolation and culture of dermal fibroblasts

Skin tissues were collected from Hsd: Athymic Nude-Foxn1nu (n = 9) (Harlan Sprague–Dawley, Indianopolis,IN) and C57BL/6 J (n = 9) (B6; Jackson Laboratory (Bar Harbor, ME) mice during three independent experiments. Tissues were minced and digested with collagenase class I (2 mg/ml; Worthington Biochemical Corp., Freehold, NJ) in a shaking water bath at 37°C for 1 hour. Dissociated dermal fibroblasts were filtered through a 100 μm cell strainer (Becton Dickinson Labware, NJ) and centrifuged at 360xg for 5 min. Pelleted cells were resuspended for 1 min in red blood cell lysing buffer (Sigma Co. St. Louis, MO) and were centrifuged at 360 × g for 5 min to remove erythrocyte contamination. Cells were stored in liquid nitrogen at concentration 2×10^6^/vial.

Thawed dermal fibroblasts (p = 0) from nude and wild type mice were plated on collagen I coated 6-well plates at density 0.2 x106 cells per well (p = 1) in Dulbecco’s Modified Eagle Medium (DMEM/F12; Life Technologies, New York, NY) supplemented with 15% fetal bovine serum (FBS - Life Technologies, New York, NY) and antibiotics. Dermal fibroblasts at 80-90% confluency were washed with DMEM/F12 media without FBS and then incubated for 24 hours in DMEM/F12 media with 5% FBS and different concentrations of cyclosporin A (CsA Sigma c1832). The range of CsA treatment doses were chosen based on *in vitro*[10] and *in vivo*[9] data from human [26] and animal [9] studies. Triplicate wells were used per treatment and the experiment was repeated three times each time using cells collected from different set of animals.

The experimental animal procedures performed in these studies have been approved by the Institutional Animal Care and Use Committee at the Pennington Biomedical Research Center.

### Flow cytometry assay

Additional groups of Hsd: Athymic Nude-Foxn1nu (n = 9) (Harlan Sprague–Dawley, Indianopolis,IN) and C57BL/6 J (n = 9) (B6; Jackson Laboratory (Bar Harbor, ME) mice were used to characterize the immunophenotype of dermal fibroblasts isolated from the skin. Cells were collected from the skin according to the isolation procedure described above. Freshly isolated cells were incubated with phycoerythrin-conjugated antiSca-1, antiCD117, antiCD90, antiCD73 and antiCD44 antibodies (BD Pharmingen, San Diego, CA) as previously described [35]. For immunophynotyping with Oct3/4 antibodies (R&D Systems, Inc.) staining was performed according to intracellular staining procedure recommended by antibody supplier. The flow cytometry assay was performed using a FACScan flow cytometer (Becton Dickinson, San Jose, CA) and data were analyzed with a Macintosh G5 workstation (Apple Computer, Cupertino, CA), which contains Cellquest graphics software (Becton Dickinson, San Jose, CA) for data acquisition and analysis. FACS plots and data are representative of three separate experiments.

### RNA isolation and quantitative RT-PCR

Total RNA was extracted using Trizol (Invitrogen, Carlsbad, CA) and column-purified with RNeasy and RNase-Free DNase kits (Qiagen, Valencia, CA). cDNA synthesis was performed with 500 ng of total RNA using the High Capacity cDNA Archive Kit (Applied Biosystems, Foster City, CA). Endogenous mRNA levels for: Mmp-3, Mmp-9, Mmp-13, α1 chain of type I collagen (col1a1), α1 chain of type III collagen (col3a1) and the housekeeping gene hypoxanthine phosphoribosyltransferase 1 (Hprt1) were measured with Applied Biosystems Taqman^®^ Gene Expression Assays (Applied Biosystems, Foster City, CA). Reactions were performed in MicroAmp Optic 384-well Reaction Plates (Applied Biosystems) using the ABI Prism 7900 Sequence Detection System (Perkin Elmer, Boston, MA) under the following incubation conditions: 2 min at 48°C, 10 min at 95°C, 40 cycles of 15 s at 95°C and 1 min at 60°C. Each run included a standard curve with aliquots from a RNA pool isolated from skin tissues, a non-template control, and minus reverse transcriptase control that were analyzed in duplicate. Expression levels for each gene estimated from the standard curve were normalized to Hprt1 and multiplied by 10.

### Protein isolation and western blot analysis

Total cell lysates were prepared by adding 400 μl of RIPA buffer containing protease inhibitor cocktail (Sigma-Aldrich, St. Louis, MO) and phosphatase inhibitor cocktails I and II (Sigma-Aldrich, St. Louis, MO). Total protein (40 μg) was separated on 12% SDS–polyacrylamide gels and transferred onto PVDF membranes (Millipore, Billerica, MA). The blots were then incubated with antibodies against PKCα, ß, ε (Cell Signaling Technology), phospho-protein kinase C antibody that detect PKCα, ß I, ß II, δ, ε and η isoforms (PKC (pan) (ßII Ser660; Cell Signaling Technology) and against β actin (Abcam Inc.). Bands were visualized using the Odyssey imaging system (LI-COR Bioscience, Lincoln, NB) with fluorescent (IRDye800TM or Alexa Fluor^®^ 680) labeled secondary antibodies according to manufacturer’s protocol.

### Wound migration assay

Dermal fibroblasts from nude and wild type mice at passage 1 were used for wound migration assay. To control cells proliferation capacity confluent dermal fibroblasts were pre-treated for 3 hours with mitomycin (10 μg/ml). Next, cells were washed with DMEM/F12 with 5% of FBS. To evaluate wound migration, confluent dermal fibroblast cultures were scraped throughout middle part of the entire 35 mm plate with the aid of 200 μl pipette tip to create a wound. Cells were cultured in 5% FBS containing DMEM/F12 with two different concentrations of cyclosporin A: 100 ng/ml or 1000 ng/ml. Cultures were photographed at 6, 12, 24 and 36 hours after wounding. Distance between post-wounded edges was visualized with camera mounted microscope and measured with Metamorph software. Triplicate wells were used per treatment and the experiment was repeated three times.

### Statistical analysis

All data are expressed as a mean ± SEM. Student’s *t* test (Microsoft Excel) was used for analyzing differences between experimental groups as indicated in the figure legends. A value of p < 0.05 was considered significant.

## Abbreviations

CsA: Cyclosporine A; MMPs: Matrix metalloproteinases.

## Competing interests

Authors declare non-competing interests.

## Authors’ contributions

BGK conception and design of the study; carried out the molecular studies and wound migration assay; acquisition, analysis and interpretation of data; writing and approving of the final version of the manuscript. HKB carried out the Western Blot analysis; helped to draft the manuscript; approved the final version of the manuscript.
